# Cost‐effectiveness analysis of first‐line treatments for advanced epidermal growth factor receptor‐mutant non‐small cell lung cancer patients

**DOI:** 10.1002/cam4.3733

**Published:** 2021-02-24

**Authors:** Wen‐Qian Li, Ling‐Yu Li, Jin Chai, Jiu‐Wei Cui

**Affiliations:** ^1^ Department of Cancer center the First Hospital of Jilin University Changchun China; ^2^ Department of pharmacy the Second Hospital of Jilin University Changchun China

**Keywords:** cost‐effectiveness, epidermal growth factor receptor, first‐line therapy, non‐small cell lung cancer

## Abstract

**Objectives:**

Recent studies showed prolonged survival for advanced epidermal growth factor receptor (EGFR)‐mutant non‐small cell lung cancer (NSCLC) patients treated with both monotherapies and combined therapies. However, high costs limit clinical applications. Thus, we conducted this cost‐effectiveness analysis to explore an optimal first‐line treatment for advanced EGFR‐mutant NSCLC patients.

**Materials and Methods:**

Survival data were extracted from six clinical trials, including ARCHER1050 (dacomitinib vs. gefitinib); FLAURA (osimertinib vs. gefitinib/erlotinib); JO25567 and NEJ026 (bevacizumab +erlotinib vs. erlotinib); NEJ009 (gefitinib +chemotherapy vs. gefitinib); and NCT02148380 (gefitinib +chemotherapy vs. gefitinib vs. chemotherapy) trials. Cost‐related data were obtained from hospitals and published literature. The effect parameter (quality‐adjusted life year [QALY]) was the reflection of both survival and utility. Incremental cost‐effectiveness ratio (ICER), average cost‐effectiveness ratio (ACER), and net benefit were calculated, and the willingness‐to‐pay (WTP) threshold was set at $30828/QALY from the perspective of the Chinese healthcare system. Sensitivity analysis was performed to explore the stability of results.

**Results:**

We compared treatment groups with control groups in each trial. ICERs were $1897750.74/QALY (ARCHER1050), $416560.02/QALY (FLAURA), ‐$477607.48/QALY (JO25567), ‐$464326.66/QALY (NEJ026), ‐$277121.22/QALY (NEJ009), ‐$399360.94/QALY (gefitinib as comparison, NCT02148380), and ‐$170733.05/QALY (chemotherapy as comparison, NCT02148380). Moreover, ACER and net benefit showed that the combination of EGFR‐TKI with chemotherapy and osimertinib was of more economic benefit following first‐generation EGFR‐TKIs. Sensitivity analyses showed that the impact of utilities and monotherapy could be cost‐effective with a 50% cost reduction.

**Conclusion:**

First‐generation EGFR‐TKI therapy remained the most cost‐effective treatment option for advanced EGFR‐mutant NSCLC patients. Our results could serve as both a reference for both clinical practice and the formulation of medical insurance reimbursement.

## Graphical abstract

First‐generation EGFR‐TKI therapy was the most cost‐effective treatment. Osimertinib and dacomitinib could be cost‐effective with a 50% cost reduction. Patients receiving combination therapy had a lower QALY than EGFR‐TKI. Results could be reference for the formulation of medical insurance reimbursement.

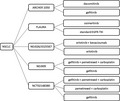

## 1 BACKGROUND

Lung cancer remains the leading cause of cancer‐related mortality worldwide. Non‐small cell lung cancer (NSCLC) is a major component, accounting for approximately 85% of cases.^1^, ^2^, ^3^ However, the 5‐year survival rate was poor and ranged 10%–20% in most countries.^4^ Epidermal growth factor receptor (EGFR) mutations are a common molecular therapeutic target occurring in approximately 40%–60% of Asian patients and 10%–20% of Caucasian patients with lung adenocarcinomas.^5^

Currently, first‐generation EGFR‐tyrosine kinase inhibitors (EGFR‐TKIs, [gefitinib, erlotinib, and icotinib]) and second‐generation EGFR‐TKI (afatinib)‐targeted therapy have demonstrated improved efficacy over conventional chemotherapy for advanced EGFR‐mutant patients and is generally accepted as the standard first‐line treatment, followed by sequential chemotherapy or third‐generation EGFR‐TKIs with EGFR T790 M mutation after disease progression.^6^, ^7^, ^8^ However, the median progression‐free survival (PFS) for first‐generation EGFR‐TKIs was less than a year.^9^ In addition, considering the impact of drug resistance to EGFR‐TKI, adverse events (AEs), physical state, psychology, and costs of drugs, the proportion of patients who benefit from sequential treatment strategies is limited.^10^, ^11^

Thus, first‐line treatment strategies for EFGR‐mutant NSCLC patients were further explored and remained challenging. Current studies have mainly focused on monotherapies including second‐ and third‐generation EGFR‐TKIs (dacomitinib and osimertinib) as well as combined therapies including combination of EGFR‐TKIs with chemotherapy and anti‐angiogenic therapy, respectively. However, despite the efficacy of multiple treatment strategies, high costs limit their clinical application.^12^, ^13^

Optimal treatment decisions should be made after balancing symptom burdens, survival outcomes, tolerability, quality of life, costs, and reimbursement issues comprehensively.^14^ Therefore, we conducted a cost‐effectiveness analysis that measured different treatment schemes via multiple dimensions from the perspective of the Chinese health system, aiming to provide guidance for treatment decisions in clinical practice. Considering the accessibility and comparability of data, six clinical trials were extracted in order to represent different first‐line treatment strategies for advanced EGFR‐mutant NSCLC patients, including two phase 3 clinical trials. These trials include ARCHER 1050 trial and FLAURA trial for monotherapy; phase 2 JO25567 trial and phase 3 NEJ026 trial for the combination of targeted therapy and antiangiogenic therapy; phase 2 NCT02148380 trial and phase 3 NEJ009 trial for the combination of targeted therapy and chemotherapy.^15^, ^16^, ^17^, ^18^, ^19^, ^20^, ^21^, ^22^, ^23^

## 2 METHODS

### 2.1 Clinical data

**FIGURE 1:**
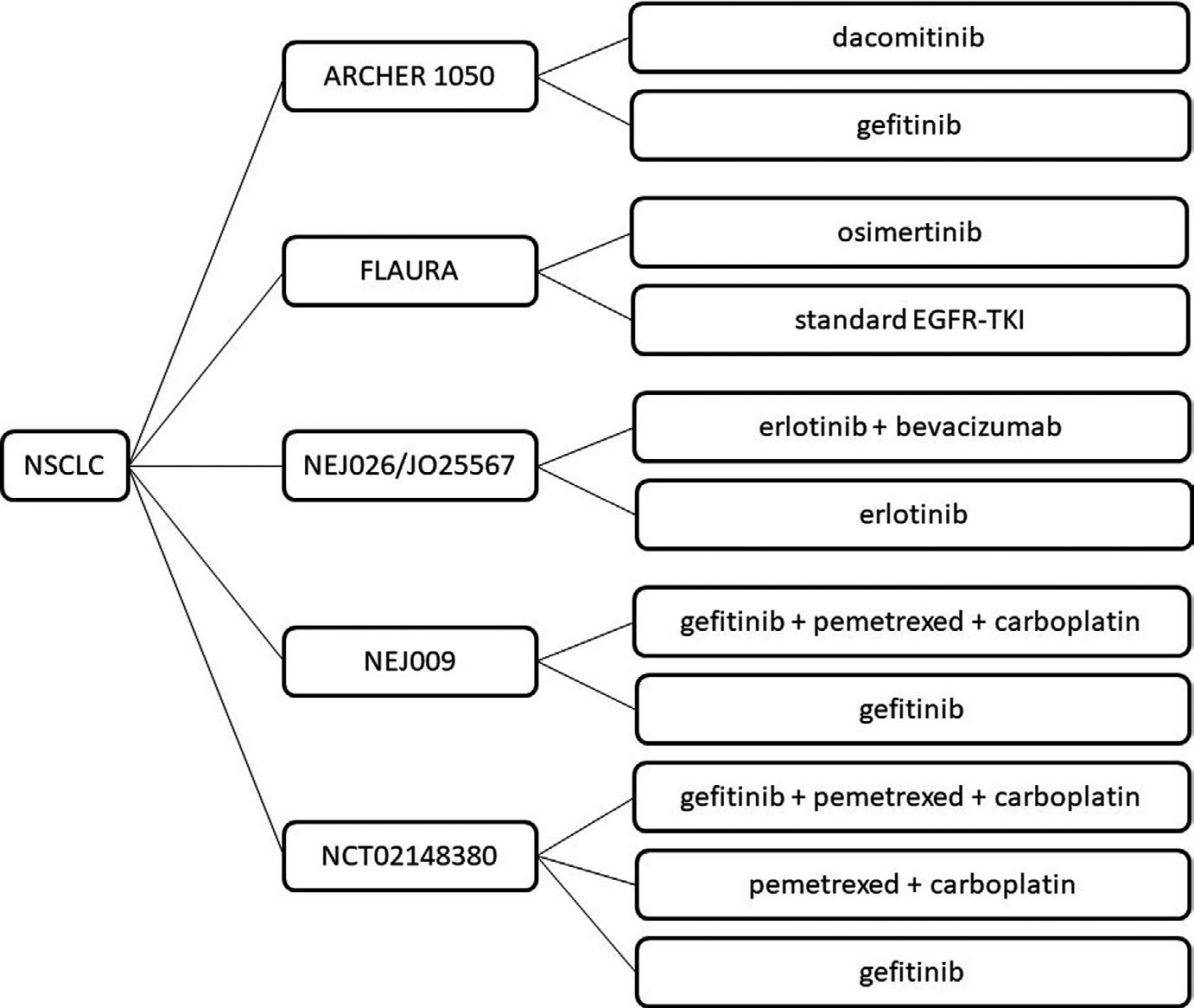
Schematic diagram of treatment strategies in clinical trials. NSCLC: non‐small cell lung cancer, EGFR‐TKI: epidermal growth factor receptor‐tyrosine kinase inhibitor

Six clinical trials exploring first‐line treatments for advanced EGFR‐mutant NSCLC patients were included (Figure 1). For monotherapy, ARCHER 1050 trial demonstrated superior survival outcomes for dacomitinib (45 mg/d) comparing with gefitinib (250 mg/d) (median PFS: 14.7 months vs. 9.2 months, *p* < 0.0001; median overall survival [OS]: 34.1 months vs. 26.8 months, *p* = 0.0438).^15^, ^16^ FLAURA trial showed significant prolonged PFS (median PFS: 18.9 months vs. 10.2 months, *p* < 0.001) and OS (median OS: 38.6 months vs. 31.8 months, *p* = 0.0462) for osimertinib (80 mg/d) compared with first‐generation EGFR‐TKIs (gefitinib, 250 mg/d or erlotinib, 150 mg/d).^17^, ^18^ Both JO25567 trial and NEJ026 trial explored the efficacy of combined bevacizumab (15 mg/kg, d1) and erlotinib (150 mg/d) compared with erlotinib (150 mg/d), superior PFS was showed in both trials (median PFS: JO25567, 16.4 months vs. 9.8 months, *p* = 0.0005; NEJ026, 16.9 months vs. 13.3 months, *p* = 0.016), while neither showed OS benefit for combined therapy (median OS: JO25567, 47.0 months vs. 47.4 months, *p* = 0.3267; NEJ026, 50.7 months vs. 46.2 months, *p* = 0.973).^19^, ^20^, ^21^ Besides, NEJ009 trial and NCT02148380 trial explored the efficacy of the combination of targeted therapy and chemotherapy (pemetrexed, 500 mg/m^2^, d1 + carboplatin, area under curve [AUC] 5, d1 + gefitinib, 250 mg/d) compared with gefitinib (250 mg/d), where significant survival benefit was shown for both PFS (median PFS: NEJ009, 20.9 months vs. 11.9 months, *p* < 0.001; NCT02148380, 17.5 months vs. 11.9 months, *p* = 0.003) and OS analyses (median OS: NEJ009, 50.9 months vs. 38.8 months, *p* = 0.021; NCT02148380, 32.6 months vs. 25.8 months, *p* = 0.001).^22^, ^23^ NCT02148380 trial also set chemotherapy (pemetrexed, 500 mg/m^2^, d1 + carboplatin, AUC 5, d1) as comparison and demonstrated both superior PFS and OS benefit for the combination group (median PFS: 17.5 months vs. 5.7 months, *p* < 0.001; median OS: 32.6 months vs. 24.3 months, *p* = 0.016).^23^ The detailed inclusion criteria, administration, survival outcomes, and AE rates are listed in Table 1.

**TABLE 1:** Characteristics of clinical trials

Trial	ARCHER 1050		FLAURA		JO25567		NEJ026		NEJ009		NCT02148380		
	dacomitinib	gefitinib	osimertinib	EGFR‐TKI	bevacizumab+erlotinib	erlotinib	bevacizumab+erlotinib	erlotinib	gefitinib +carboplatin + pemetrexed	gefitinib	pemetrexed +carboplatin + gefitinib	pemetrexed +carboplatin	gefitinib
Phase	3		3		2		3		3		2		
Country	international		international		Japan		Japan		Japan		China		
Inclusion criteria	advanced (stage IIIB/IV or recurrent) EGFR‐mutated (ex19del or L858R mutation, with or without T790 M) NSCLC, aged≥18 years, ECOG 0–1, newly diagnosed, without CNS metastases		advanced (locally advanced or metastatic) EGFR‐mutated (ex19del or L858R mutation) NSCLC, previously untreated, neurologically stable CNS metastases		advanced (stage IIIB/IV or recurrent) non‐squamous EGFR‐mutated (ex19del or L858R mutation, without T790 M) NSCLC, age≥20 years, ECOG 0–1, no previous chemotherapy for advanced disease, without CNS metastases		advanced (stage IIIB/IV or recurrent) non‐squamous EGFR‐mutated (ex19del or L858R mutation, without T790 M) NSCLC, age≥20 years, ECOG 0–2, no previous chemotherapy for advanced disease, asymptomatic brain metastasis		advanced (stage IIIB/IV or relapsed) non‐squamous EGFR‐mutated (ex19del, L858R, G719A, G719C, G719S, and L861Q, without T790 M) NSCLC, age 20–75 years, ECOG 0–1, newly diagnosed, chemotherapy naive, without CNS metastases		advanced (locally advanced or metastatic) adenocarcinoma EGFR‐mutated (ex19del or L858R mutation) NSCLC, age≥18 years, ECOG 0–1, did not received systemic anticancer therapy for advanced disease; neurologically stable CNS metastases		
Cycle length	28 days		21 days		21 days		21 days		21 days		28 days		
Administration	dacomitinib, 45 mg/d	gefitinib, 250 mg/d	osimertinib, 80 mg/d	gefitinib, 250 mg/d or erlotinib, 150 mg/d	bevacizumab, 15 mg/kg, d1 + erlotinib, 150 mg/d	erlotinib, 150 mg/d	bevacizumab, 15 mg/kg, d1 + erlotinib, 150 mg/d	erlotinib, 150 mg/d	pemetrexed, 500 mg/m2, d1 + carboplatin, AUC 5, d1 + gefitinib, 250 mg/d (up to 6 cycles), followed by maintenance pemetrexed +gefitinib	gefitinib, 250 mg/d	pemetrexed, 500 mg/m2, d1 + carboplatin, AUC 5, d1 + gefitinib, 250 mg/d, d5–21 (up to 6 cycles), followed by maintenance pemetrexed +gefitinib	pemetrexed, 500 mg/m2, d1 + carboplatin, AUC 5, d1 (up to 6 cycles), followed by maintenance pemetrexed	gefitinib, 250 mg/d
Median PFS	14.7 months	9.2 months	18.9 months	10.2 months	16.4 months	9.8 months	16.9 months	13.3 months	20.9 months	11.9 months	17.5 months	5.7 months	11.9 months
Median OS	34.1 months	26.8 months	38.6 months	31.8 months	47.0 months	47.4 months	50.7 months	46.2 months	50.9 months	38.8 months	32.6 months	24.3 months	25.8 months
ORR	75%	72%	80%	76%	69%	64%	72%	66%	84%	67%	82.50%	32.50%	65.90%
AE≥3	63%	41%	34%	45%	91%	53%	88%	46%	65.30%	31%	37.5%	17.50%	12.20%

Abbreviations: NSCLC, non‐small cell lung cancer; EGFR, epidermal growth factor receptor; EGFR‐TKI, epidermal growth factor receptor‐tyrosine kinase inhibitor; ECOG, Eastern Cooperative Oncology Group; CNS, central nervous system; AUC, area under curve; PFS, progression‐free survival; OS, overall survival; ORR, objective response rate; AE, adverse events.

### 2.2 Markov model

To simulate the disease transformation of patients, a Markov model was established by Treeage Pro. Cycle length was set according to clinical trials with a 28‐day cycle length for the ARCHER 1050 trial, NCT02148380 trial, and a 21‐day cycle length for the other trials. Evaluation was conducted in both the 5‐year and 10‐year horizons. Patients were divided into three mutually independent Markov states: PFS, progressive disease (PD), and death. All patients were first in the PFS state with a probability of 1, after which patients could transfer to other states on the basis of transition probabilities. The calculation of transition probabilities was based on the PFS and OS Kaplan‐Meier curves in each trial using GetData Graph Digitizer and Matlab software. Progressive or survival probability P was calculated using the following formula: P = 1 − Exp(−r × t), where r was the progressive or survival rate at time t. According to Weibull model, transition probability Pt was calculated based on the formula: Pt =1–Exp [λ(t − u)^γ^ – λt^γ^], where u represented for the cycle length, λ and γ represented for the scale and shape parameter.^24^

### 2.3 Cost and utility

Cost parameters were obtained from local hospitals and published literature. Direct medical costs, including costs of drugs, examinations, follow‐up, supportive care, AE ≥grade 3, progressive disease (PD), and terminal care were calculated.^25^, ^26^ We assumed a typical patient with a height of 1.64 m, a weight of 65 kg, and a body surface area (BSA) of 1.72 m^2^ to calculate the costs of intravenous drugs. Costs were measured in U.S. dollars at an exchange rate of 7.0459.

Quality‐adjusted life year (QALY) was measured as an effect parameter, which represented a combination of survival and utility. Utility is the reflection of a patient's quality of life, which ranges from 0 to 1. The utilities of administration methods (including oral and intravenous administration) as well as utilities of different EGFR‐TKIs, were distinguished.^15^, ^27^ In addition, utilities of PD state and AE ≥grade 3 were included.^27^, ^28^ Both costs and utilities are displayed in Table 2, and the discount rate was set at 3%.

**TABLE 2:** Parameters

Parameters	value	Specification
Cost		
osimertinib	2171.48	80 mg*30
gefitinib	22.65	0.25 g*1
erlotinib	10.08	0.15 g*1
bevacizumab	212.89	100 mg*1
carboplatin	7.34	100 mg*1
pemetrexed	190.61	0.2 g*1
dacomitinib	803.30	15 mg*30
CT Scan‐Lung	53.24	once
CT Scan‐Abdomen	50.81	once
MRI Scan‐brain	88.70	once
electrocardiograph	3.69	once
echocardiography	47.18	once
routine blood test	3.12	once
blood biochemical examination	24.55	once
routine urine test	4.26	once
coagulation test	9.08	once
artery blood gas	21.29	once
follow‐up	55.60	cycle
supportive care	337.50	cycle
AE	507.40	cycle
average PD	276.75	week
average terminal care	1412.92	week
Utility		
gefitinib	0.8000	
erlotinib	0.8100	
first‐generation TKI	0.8050	
dacomitinib	0.8270	
osimertinib	0.8400	
intravenous therapy	0.7600	
PD	0.7000	
AE	−0.0731	

Abbreviations: CT, computed tomography; MRI, magnetic resonance imaging; TKI, tyrosine kinase inhibitor; PD, progressive disease; AE, adverse events.

### 2.4 Outcomes

We conducted cost‐effectiveness analyses for various treatment groups and comparative groups in different clinical trials using both the 5‐year horizon and 10‐year horizon. The willingness‐to‐pay (WTP) threshold was set at 3 per gross national product (GDP), $30828/QALY in China. The primary result was expressed as incremental cost‐effectiveness ratio ([ICER], ICER=incremental cost/ incremental effect), representing the results of intra‐group cost‐effectiveness analyses. Besides, we further explored the ideal percentage of cost adjustments based on the WTP threshold, incremental effect, and current costs using a positive ICER. Secondary results included the average cost‐effectiveness ratio ([ACER], ACER =cost/ effect) and net benefit (net benefit=QALY* willingness‐to‐pay [WTP] threshold‐cost), focusing on inter‐group cost‐effectiveness analyses.

To detect the influence of various parameters and the stability of results, both one‐way sensitivity analysis and probabilistic sensitivity analysis were performed by Treeage. In the one‐way sensitivity analysis, cost parameters were assumed with a 30% range and a 20% range for both utilities and probabilities ^28^ (Table S1). Results are shown in Tornado Diagrams. For probabilistic sensitivity analysis, a Monte Carlo simulation with 1000 iterations was conducted, and cost parameters were assumed to fit the gamma distribution, while other parameters fitted the beta distribution,^29^ and the results would be shown in cost‐effectiveness acceptability curves.

## 3 RESULTS

First, in the primary intra‐group cost‐effectiveness analyses, the ICER was $1897750.74/QALY for monotherapies, when comparing dacomitinib with gefitinib based on the ARCHER 1050 trial. Based on the FLAURA trial, osimertinib demonstrated an ICER of $416560.02/QALY compared with first‐generation EGFR‐TKIs. As for the combined therapies, when comparing bevacizumab plus erlotinib with erlotinib alone, both JO25567 trial (ICER: ‐$477607.48/QALY) and NEJ026 trial (ICER: ‐$464326.66/QALY) showed negative ICER since the QALY of the combination was lower than that in the erlotinib group. Besides, in terms of the combination of chemotherapy and gefitinib, the ICERs were also negative due to lower QALY. Based on the NEJ009 trial, the ICER of the combination of gefitinib and chemotherapy was ‐$277121.22/QALY compared with gefitinib. In the NCT02148380 trial, the ICER of the combination of gefitinib and chemotherapy was ‐$399360.94/QALY and ‐$170733.05/QALY when compared with chemotherapy and gefitinib, respectively. In addition, for comparisons with positive ICERs, further cost adjustments were calculated, compared with first‐generation EGFR‐TKIs, osimertinib could be cost‐effective with a cost reduction of 49.75% based on the FLAURA trial, while dacomitinib could achieve economic benefit with a cost reduction of 51.98% in the ARCHER 1050 trial.

As for the secondary inter‐group cost‐effectiveness analyses, results of ACER showed except for first‐generation EGFR‐TKIs with a range of ACER from $18556.46/QALY to $27416.56/QALY and chemotherapy with an ACER of $27957.76/QALY, the combination of chemotherapy and gefitinib demonstrated favorable ACER of $36485.62/QALY based on NCT02148380 trial, followed by an ACER of $40937.92/QALY for osimertinib based on FLAURA trial, an ACER of $50214.97/QALY for dacomitinib based on ARCHER 1050 trial, an ACER of $55239.44/QALY for the combination of chemotherapy and gefitinib based on NEJ009 trial, while bevacizumab combined with erlotinib had the highest ACER ($74573.71/QALY, NEJ026 trial; $75160.74/QALY, JO25567 trial). The net benefit was consistent with the results. Detailed results of cost parameters, QALY parameters, incremental parameters, ACER, net benefit, and ICER in both 5‐year and 10‐year horizons are listed in Table 3. The parameters of the transition probability are listed in Table S2. The cost‐effectiveness analysis curves are shown in Figure S1.

**TABLE 3:** Results of cost‐effectiveness analyses

	ARCHER 1050		FLAURA		JO25567		NEJ026		NEJ009		NCT02148380		
	dacomitinib	gefitinib	osimertinib	EGFR‐TKI	bevacizumab+erlotinib	erlotinib	bevacizumab+erlotinib	erlotinib	gefitinib+carboplatin+ pemetrexed	gefitinib	pemetrexed+carboplatin+gefitinib	pemetrexed+carboplatin	gefitinib
5‐year													
cost	91798.60	43296.13	63933.01	29583.24	99859.26	28690.10	99392.65	27601.34	75379.85	40830.75	62569.47	48901.81	39191.20
QALY	1.83	1.80	1.56	1.48	1.33	1.48	1.33	1.49	1.36	1.49	1.71	1.75	1.85
ACER	50214.97	24019.32	40937.92	19998.87	75160.74	19416.41	74573.71	18556.46	55239.44	27416.56	36485.62	27957.76	21163.42
net benefit	−35441.56	12273.01	−15788.73	16018.95	−58900.90	16862.01	−58304.76	18252.99	−33311.89	5080.57	−9702.30	5020.42	17897.22
IC	48502.47		34349.77		71169.17		71791.31		34549.09			13667.67	23378.27
IE	0.03		0.08		−0.15		−0.15		−0.12			−0.03	−0.14
ICER	1897750.74		416560.02		−477607.48		−464326.66		−277121.22			−399360.94	−170733.05
10‐year													
cost	107379.86	50644.91	69941.25	32363.39	109243.75	31386.31	108733.29	30195.23	82463.82	44667.91	73189.59	57202.06	45843.25
QALY	2.14	2.11	1.71	1.62	1.45	1.62	1.46	1.63	1.49	1.63	2.01	2.05	2.17
ACER	50214.97	24019.32	40937.92	19998.87	75160.74	19416.41	74573.71	18556.46	55239.44	27416.56	36485.62	27957.76	21163.42
net benefit	−41457.16	14356.15	−17272.51	17524.36	−64436.23	18446.65	−63784.07	19968.35	−36442.45	5558.03	−11349.10	5872.56	20934.97
IC	56734.94		37577.86		77857.44		78538.05		37795.92			15987.53	27346.34
IE	0.03		0.09		−0.16		−0.17		−0.14			−0.04	−0.16
ICER	1897750.74		416560.02		−477607.48		−464326.66		−277121.22			−399360.94	−170733.05

Abbreviations: EGFR‐TKI, epidermal growth factor receptor‐tyrosine kinase inhibitor; QALY, quality‐adjusted life year; ACER, average cost‐effectiveness ratio; IC, incremental cost; IE, incremental effect; ICER, incremental cost‐effectiveness ratio.

**FIGURE 2:**
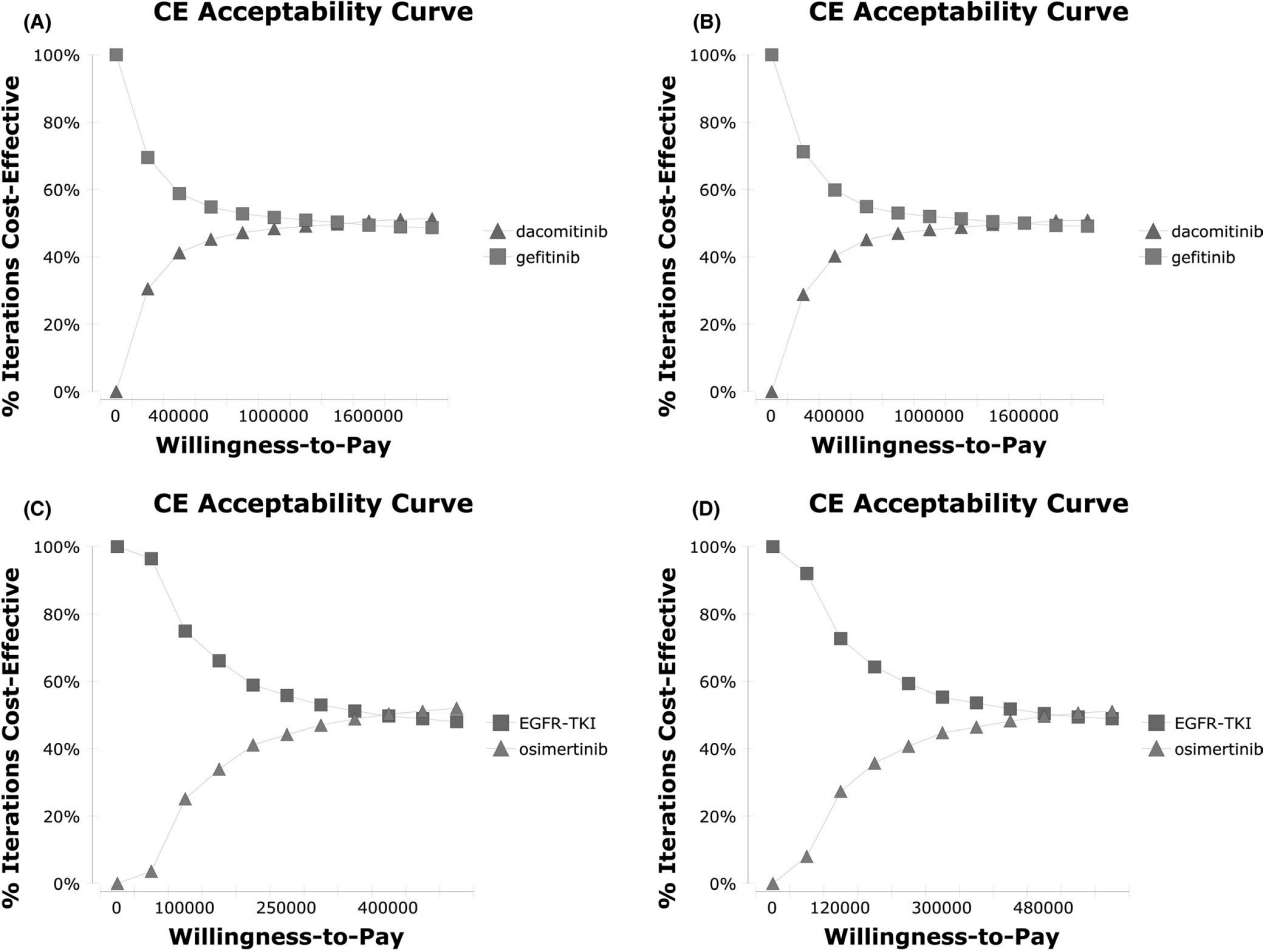
Cost‐effectiveness acceptability curve for comparison among various treatment regimens. A: ARCHER 1050 trial in 5‐year horizon, B: ARCHER 1050 trial in 10‐year horizon, C: FLAURA trial in 5‐year horizon, D: FLAURA trial in 10‐year horizon. EGFR‐TKI: epidermal growth factor receptor‐tyrosine kinase inhibitor, CE: cost‐effectiveness

Finally, in one‐way sensitivity analyses, the utility of the combination group showed a greater impact in the NCT02148380 and NEJ009 trials over the 5‐year horizon. Meanwhile, in other cases, utility parameters in first‐generation EGFR‐TKI groups had a greater impact on the results. Results are shown in Figure S2 and Figure S3. For probabilistic sensitivity analyses, cost‐effectiveness acceptability curves showed that at a WTP threshold of $1600000/QALY, dacomitinib could be cost‐effective based on the ARCHER1050 trial, and at a WTP threshold of approximately $350000/QALY ‐ $500000/QALY, osimertinib could be cost‐effective (Figure 2).

## 4 DISCUSSION

Despite the rapid development of anti‐tumor therapies, the prognosis of advanced NSCLC patients remains poor. Thus, choices of first‐line treatment strategies are of high clinical concern, and it is urgent to weigh treatment options from multiple dimensions in order to provide guidance for clinical practice.^30^ To the best of my knowledge, this is the first cost‐effectiveness analysis to comprehensively evaluate first‐line treatment strategies from multiple dimensions for advanced EGFR‐mutant patients. Six clinical trials were extracted representing targeted monotherapy (ARCHER 1050 trial, FLAURA trial), the combination of targeted therapy and antiangiogenic therapy (JO25567 trial, NEJ026 trial), and the combination of targeted therapy and chemotherapy (NCT02148380 trial, NEJ009 trial). We analyzed the intra‐group cost‐effectiveness ratio between various treatment regimens and corresponding control groups based on clinical trials. Results showed that with the current WTP threshold ($30828/QALY), none of the emerging therapies was cost‐effective compared with standard first‐generation EGFR‐TKIs for advanced EGFR‐mutant patients. As for inter‐group comparison, when considering ACER and net benefit, the combination of EGFR‐TKI with chemotherapy and osimertinib showed more economic benefit following the application of first‐generation EGFR‐TKIs. Probabilistic sensitivity analysis suggested that monotherapy could be cost‐effective at a higher WTP threshold.

Common methods of cost‐effectiveness analyses included both the construction of the Markov model and the calculation of QALY through the area under survival curves (AUC).^31^, ^32^ However, the AUC calculation was highly influenced by the heterogeneity among trials, especially different follow‐up periods, while the calculation of transition probabilities and horizon settings in the Markov model could reduce the heterogeneity. Since, the current study involved inter‐group comparisons among multiple trials, we chose the Markov model method. In addition, Treeage software is a commonly used and specialized software for cost‐effectiveness analyses. Thus, we built the decision tree and Markov model according to relevant literature.^33^, ^34^, ^35^

The results of the cost‐effectiveness analysis were mainly affected by the following aspects. First, the prolongation of survival period was an important influencing factor. Although the prolongation of PFS was significant in all six trials, only the investigations of monotherapy (dacomitinib and osimertinib) and the combination therapy of targeted therapy with chemotherapy extended the survival benefit in OS analysis. Therefore, it was suggested that although clinical trials were often compared with PFS, the OS benefit was important in cost‐effectiveness analysis. Second, the patient's quality of life was also an important factor in measuring economic benefits. Considering the lower health state utilities of intravenous administration than oral administration as well as the disutility of AEs, intravenous combination therapy had lower health state utilities than oral monotherapies. As a result, only patients administered EGFR‐TKI monotherapy had higher QALYs than the standard first‐generation EGFR‐TKI group. Third, high costs also affected the clinical application of drugs. In China, first‐generation EGFR‐TKIs, afatinib, and chemotherapeutic drugs were covered by medical insurance reimbursement, while dacomitinib, first‐line osimertinib, and bevacizumab were still under negotiation. In addition to clinical data, adjustments to the costs of drugs and health insurance policies are also important factors for the cost‐effectiveness of various treatment regions. Thus, we further explored the ideal cost concessions for treatment regimens with high ICERs in order to achieve economic benefits. The results showed that with a cost reduction of 49.75% and 51.98%, osimertinib and dacomitinib could be cost‐effective, respectively.

Previous cost‐effectiveness analyses have mainly focused on different generations of EGFR‐TKI monotherapy. Studies have demonstrated that first‐generation EGFR‐TKIs are cost‐effective compared with conventional chemotherapy.^36^ In the comparison between afatinib and first‐generation EGFR‐TKIs, current cost‐effectiveness studies showed controversial results, the prognosis and economic benefits were relatively similar, and treatment decisions could be relied on personal characteristics of patients in a comprehensive view.^37^, ^38^, ^39^ However, none of the cost‐effectiveness analyses exploring first‐line osimertinib showed economic benefit, similar to our results.^31^, ^40^, ^41^ Besides, little or no previous studies have explored the cost‐effectiveness of combination therapy of EGFR‐TKIs.

This study had several limitations. First, the inter‐group comparison was indirect because the heterogeneity among studies could not be neglected. Differences were found in the inclusion criteria, FLAURA, NCT02148380, and NEJ026 trials included patients with neurologically stable central nervous system (CNS) metastases, while patients with CNS metastases were excluded from other trials. Clinical characteristics of eligible patients, subsequent treatments after disease progression, items and frequency of examinations, proportions of cross‐over patients, and follow‐up time also varied. Further head‐to‐head trials are expected to explore the optimal treatment strategy for advanced EGFR mutant NSCLC patients, including the conventional strategy that sets first‐generation EGFR‐TKIs as first‐line administration followed by second‐line osimertinib for T790 M mutation‐positive patients. Second, several phase 3 clinical trials were not included, mainly due to the lack of data. Phase 3 RELAY trial focused on the comparison between ramucirumab plus erlotinib versus erlotinib; however, despite the absence of OS data, ramucirumab has not yet been widely used in Chinese clinical practice.^42^ The CTONG1509 trial was the first randomized phase 3 trial comparing the combination of bevacizumab and erlotinib versus erlotinib in a Chinese population with advanced EGFR‐mutant NSCLC. However, since only PFS data from oral reports were available, we did not include this study.^43^ Cost‐effectiveness analyses could be further evaluated by updating the data. Finally, studies showed a distinction between EGFR mutation subtypes, which influenced the survival outcomes of first‐line treatments.^44^ Further cost‐effectiveness analyses should also be based on distinct grognoses of EGFR mutation subtypes for choosing the optimal treatment strategies for advanced EGFR‐mutant NSCLC patients.

## 5 CONCLUSION

Based on survival data from clinical trials, patients’ quality of life and current costs of medical resources, standard first‐generation EGFR‐TKI therapy remained the most cost‐effective treatment option for advanced EGFR‐mutant NSCLC patients comprehensively.

## CONFLICT OF INTEREST

None.

## AUTHOR CONTRIBUTIONS

Guarantor of integrity of the entire study: Jiu‐Wei Cui; study concepts and design: Jiu‐Wei Cui, Wen‐Qian Li; literature research: Wen‐Qian Li, Ling‐Yu Li; data analysis: Wen‐Qian Li, Jin Chai; manuscript preparation: Wen‐Qian Li, Jiu‐Wei Cui; manuscript editing: Jiu‐Wei Cui.

## Supporting information

Fig S1

Fig S2

Fig S3

Supplementary Material

## Data Availability

Clinical data of the current study were extracted from published clinical trials, which are listed in the references. Cost, utility, and other parameters are presented in the tables. Thus, no other data were available.
