# Supplementary figures and images for: Cost‐effectiveness analysis of first‐line treatments for advanced epidermal growth factor receptor‐mutant non‐small cell lung cancer patients
Source: Cancer Med. 2021 Feb 24;10(6):1964–74. doi: 10.1002/cam4.3733 (PMC7957173; doi:10.1002/cam4.3733)

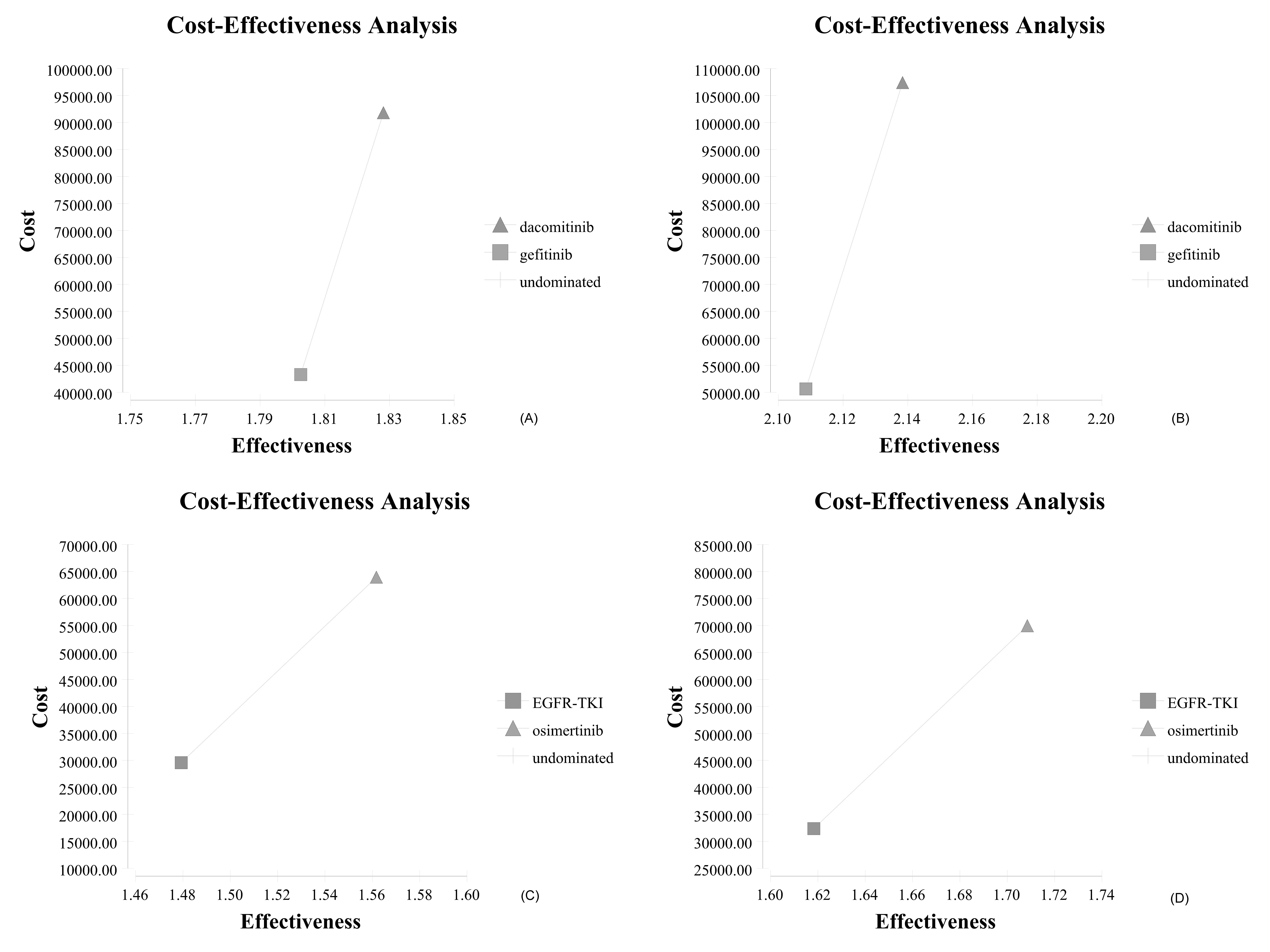

Supplement: Supplementary file 1 — Fig S1 [file CAM4-10-1964-s004.jpg]

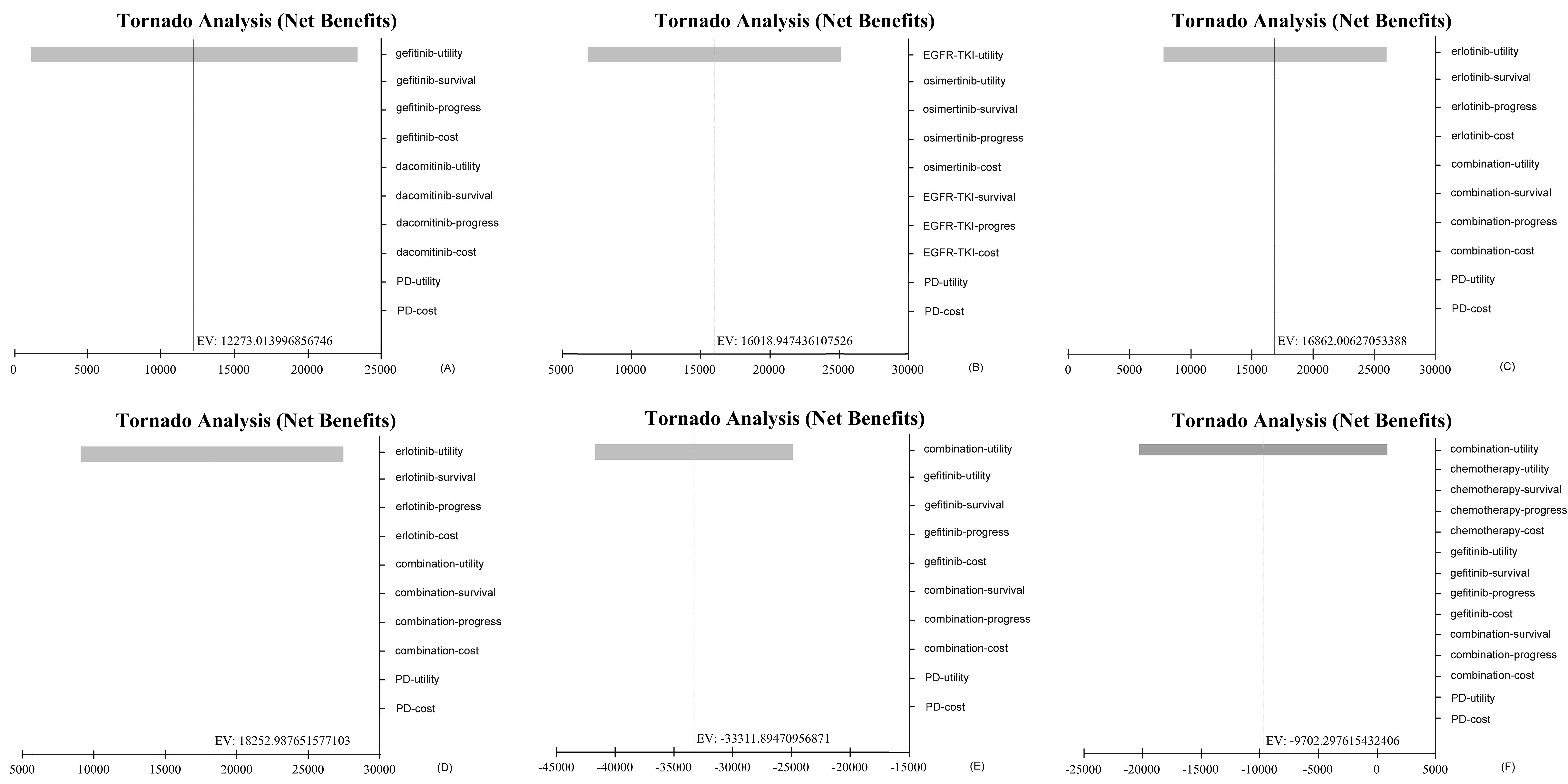

Supplement: Supplementary file 2 — Fig S2 [file CAM4-10-1964-s003.jpg]

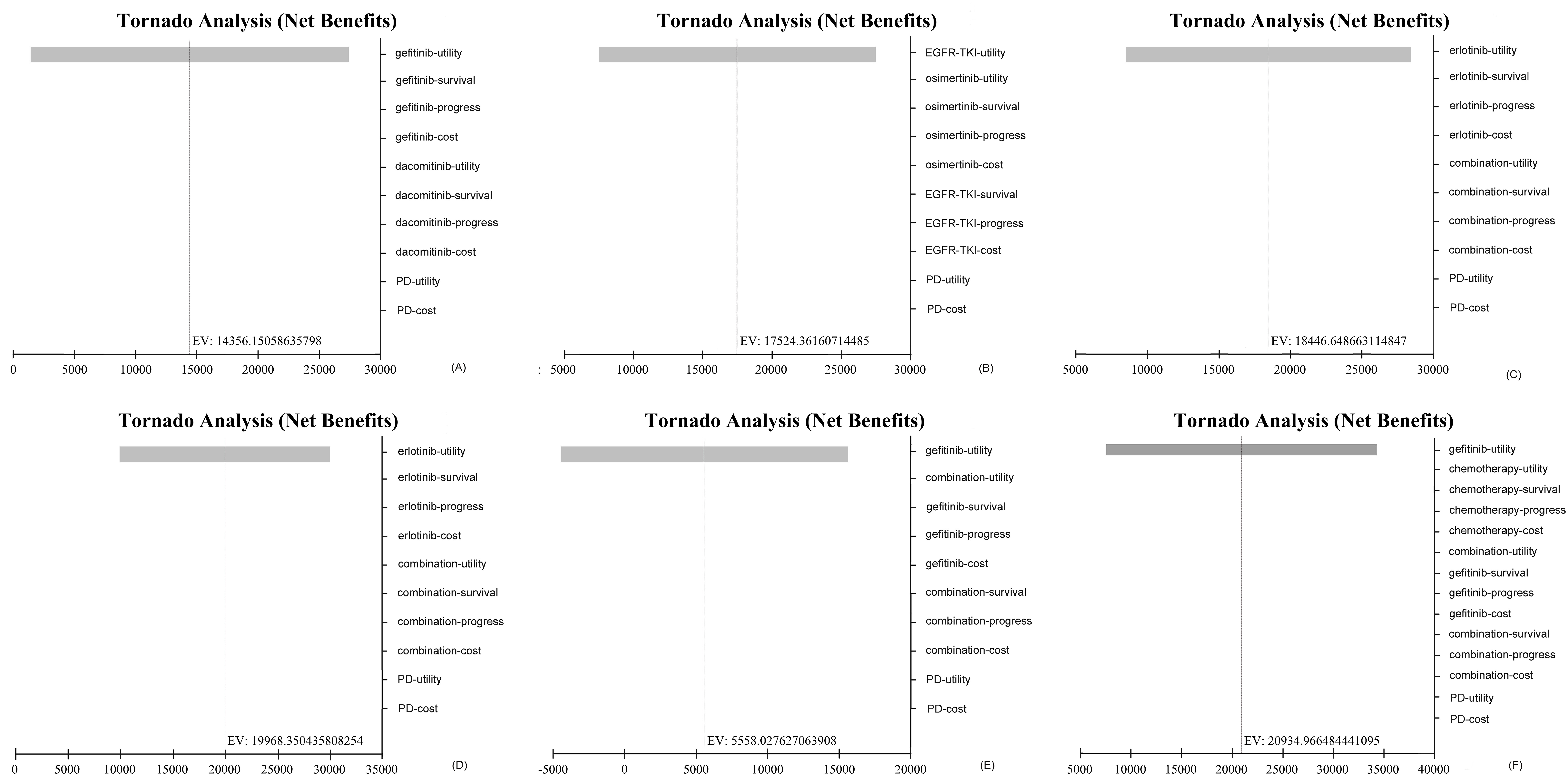

Supplement: Supplementary file 3 — Fig S3 [file CAM4-10-1964-s001.jpg]
