# Supplementary material for: Cost‐effectiveness analysis of first‐line treatments for advanced epidermal growth factor receptor‐mutant non‐small cell lung cancer patients
Source: Cancer Med. 2021 Feb 24;10(6):1964–74. doi: 10.1002/cam4.3733 (PMC7957173; doi:10.1002/cam4.3733)
Supplement: Supplementary file 4 — Supplementary Material [file CAM4-10-1964-s002.docx]

**Supplemental Figure Legend**

**Fig S1**. Cost-effectiveness analysis curves for comparison among various treatment regimens. EGFR-TKI: epidermal growth factor receptor-tyrosine kinase inhibitor.

**Fig S2.** Tornado diagrams for cost-effectiveness analysis in 5-year horizon. A: ARCHER 1050 trial, B: FLAURA trial, C: JO25567 trial, D: NEJ026 trial, E: NEJ009 trial, F: NCT02148380 trial. EGFR-TKI: epidermal growth factor receptor-tyrosine kinase inhibitor, PD: progressive disease, EV: economic value.

**Fig S3.** Tornado diagrams for cost-effectiveness analysis in 10-year horizon. A: ARCHER 1050 trial, B: FLAURA trial, C: JO25567 trial, D: NEJ026 trial, E: NEJ009 trial, F: NCT02148380 trial. EGFR-TKI: epidermal growth factor receptor-tyrosine kinase inhibitor, PD: progressive disease, EV: economic value.

**Supplemental Table 1. Sensitivity analysis**

| Trial | ARCHER 1050 | | FLAURA | | JO25567 | | NEJ026 | | NEJ009 | | NCT02148380 | | | PD | |
| --- | --- | --- | --- | --- | --- | --- | --- | --- | --- | --- | --- | --- | --- | --- | --- |
|  | dacomitinib | gefitinib | osimertinib | EGFR-TKI | bevacizumab+erlotinib | erlotinib | bevacizumab+erlotinib | erlotinib | gefitinib +carboplatin + pemetrexed | gefitinib | pemetrexed + carboplatin + gefitinib | pemetrexed + carboplatin | gefitinib | 28-day cycle | 21-day cycle |
| cost | 3511.55 | 1784.91 | 2391.57 | 1271.00 | 3625.88 | 1304.17 | 3486.04 | 1144.04 | 2473.76 | 1332.00 | 2242.10 | 1755.54 | 1395.17 | 2503.00 | 1877.25 |
| cost LL | 2458.09 | 1249.44 | 1674.10 | 889.70 | 2538.11 | 912.92 | 2440.23 | 800.83 | 1731.63 | 932.40 | 1569.47 | 1228.88 | 976.62 | 1752.10 | 1314.08 |
| cost UL | 4565.02 | 2320.39 | 3109.04 | 1652.30 | 4713.64 | 1695.42 | 4531.86 | 1487.25 | 3215.89 | 1731.60 | 2914.72 | 2282.20 | 1813.72 | 3253.90 | 2440.43 |
| utility | 0.0651 | 0.0642 | 0.0509 | 0.0483 | 0.0433 | 0.0482 | 0.0435 | 0.0485 | 0.0445 | 0.0486 | 0.0610 | 0.0623 | 0.0659 | 0.0583 | 0.0438 |
| utility LL | 0.0521 | 0.0513 | 0.0408 | 0.0386 | 0.0347 | 0.0386 | 0.0348 | 0.0388 | 0.0356 | 0.0389 | 0.0488 | 0.0498 | 0.0527 | 0.0467 | 0.0350 |
| utility UL | 0.0781 | 0.0770 | 0.0611 | 0.0579 | 0.0520 | 0.0578 | 0.0522 | 0.0582 | 0.0534 | 0.0583 | 0.0733 | 0.0747 | 0.0791 | 0.0700 | 0.0525 |
| progressive probability | 0.0470 | 0.0502 | 0.0170 | 0.0258 | 0.0153 | 0.0339 | 0.0165 | 0.0264 | 0.0145 | 0.0220 | 0.0310 | 0.1050 | 0.0530 | / | / |
| progressive probability LL | 0.0376 | 0.0401 | 0.0136 | 0.0206 | 0.0123 | 0.0271 | 0.0132 | 0.0211 | 0.0116 | 0.0176 | 0.0248 | 0.0840 | 0.0424 | / | / |
| progressive probability UL | 0.0564 | 0.0602 | 0.0204 | 0.0309 | 0.0184 | 0.0406 | 0.0198 | 0.0317 | 0.0174 | 0.0264 | 0.0372 | 0.1260 | 0.0636 | / | / |
| survival probability | 0.0202 | 0.0179 | 0.0091 | 0.0110 | 0.0042 | 0.0061 | 0.0048 | 0.0049 | 0.0051 | 0.0100 | 0.0072 | 0.0285 | 0.0164 | / | / |
| survival probability LL | 0.0161 | 0.0143 | 0.0073 | 0.0088 | 0.0034 | 0.0049 | 0.0039 | 0.0039 | 0.0041 | 0.0080 | 0.0057 | 0.0228 | 0.0131 | / | / |
| survival probability UL | 0.0242 | 0.0215 | 0.0110 | 0.0131 | 0.0051 | 0.0073 | 0.0058 | 0.0058 | 0.0062 | 0.0120 | 0.0086 | 0.0342 | 0.0197 | / | / |

EGFR-TKI: epidermal growth factor receptor-tyrosine kinase inhibitor, UL: upper limit, LL: lower limit.

**Supplemental Table 2. Results of transition probability**

| Trial | Treatment | progressive transition probability | | survival transition probability | |
| --- | --- | --- | --- | --- | --- |
|  |  | shape | scale | shape | scale |
| ARCHER 1050 | Dacomitinib | 1.9190 | 0.0481 | 1.8250 | 0.0204 |
|  | Gefitinib | 2.0250 | 0.0515 | 1.9840 | 0.0181 |
| FLAURA | Osimertinib | 1.8660 | 0.0294 | 1.7860 | 0.0154 |
|  | EGFR-TKI | 1.8870 | 0.0449 | 1.8090 | 0.0185 |
| JO25567 | Bevacizumab + erlotinib | 1.8600 | 0.0264 | 1.9240 | 0.0074 |
|  | Erlotinib | 1.8060 | 0.0579 | 1.9050 | 0.0105 |
| NEJ026 | Bevacizumab + erlotinib | 1.8380 | 0.0282 | 1.8800 | 0.0083 |
|  | Erlotinib | 1.8170 | 0.0452 | 1.9170 | 0.0085 |
| NEJ009 | Gefitinib + carboplatin + pemetrexed | 1.8510 | 0.0249 | 1.8490 | 0.0088 |
|  | Gefitinib | 1.9290 | 0.0387 | 1.7560 | 0.0167 |
| NCT02148380 | Pemetrexed + carboplatin + gefitinib | 1.9300 | 0.0315 | 2.0180 | 0.0072 |
|  | Pemetrexed + carboplatin | 1.9940 | 0.1109 | 1.8720 | 0.0289 |
|  | Gefitinib | 2.0290 | 0.0544 | 1.9870 | 0.0165 |
